# A Systematic Review of Multilevel Influenced Risk-Taking in Helicopter and Small Airplane Normal Operations

**DOI:** 10.3389/fpubh.2022.823276

**Published:** 2022-05-12

**Authors:** Matt R. Harris, Erich C. Fein, M. Anthony Machin

**Affiliations:** School of Psychology and Wellbeing, University of Southern Queensland, Toowoomba, QLD, Australia

**Keywords:** risk-taking, risk perception, weather-related decision-making, plan-continuation errors, aviation, social psychological pressure

## Abstract

The violation of aviation rules, particularly meteorological flight rules, can have fatal outcomes. Violation can sometimes be explained by intentional risk-taking, alternatively it can be the manifestation of a strategy to enhance performance and influence outcomes, such as saving time or fulfilling customer expectations. The aim of this study was to determine the types of risk-taking behavior within extant empirical research and identify multilevel antecedents related to risk-taking in the context of aviation operations, via a systematic literature review. 4,742 records were identified, which after screening resulted in the detailed consideration of 10 studies, three qualitative and seven quantitative studies, which met the eligibility criteria. Only published works were included in the review, thus the results may have been subject to publication bias, however, the types of risk taking within the research were consistent with that observed in Australian and New Zealand accident reports. The predominate risk-taking behavior was that of continuing Visual Flight Rules (VFR) flight into deteriorating conditions / Instrument Meteorological Conditions (IMC). Multilevel influences could be categorized under two overarching themes, being “continuation influence” and “acceptance of risk / normalization of deviance.” One or both themes was consistently observed across the finding in all studies, although precaution should be given to the relative frequency of the reported associations. This review indicates the value of considering the social and organizational influences on risk-taking, and suggests avenues for future research, in particular exploring the influences through a Self-Determination Theory (SDT) lens.

## Introduction

The unsafe act of violation (1) is the intentional deviation from minimum standards, operating procedures, or rules or regulations. Where precautions are not taken to mitigate a potentially negative outcome (driving at an excessive speed, etc.) the violating behavior will be judged as socially unacceptable. In contrast, where the danger is recognized and the negative outcome is seen as minor (for example, crossing the road on a “red man” when there is no traffic), the behavior will be judged as socially acceptable risk-taking (2).

“Civil Aviation Rules (the Rules) set the common minimum standards to manage risks in aviation and for entering and operating within the civil aviation system. Rules function as a combination of prescriptive standards, and performance- and risk-based requirements” (3). Thus, within the aviation context, intentionally deviating from minimum standards (violation), or knowingly pushing the human or aircraft performance limitations beyond the safety boundaries, will be judged as risk-taking behavior.

## Aviation Risk-Taking in Part 135 Type Operations

In regions with remote areas, Part 135 type operations are considered essential for transporting people and cargo.^1^ Furthermore, within the context of the novel coronavirus (COVID-19) pandemic, indications are that more people will turn to Part 135 type operations for their transportation needs, due to the perceived health benefits that come with ‘avoiding the crowds' on larger airlines.

Part 135 type operations must meet regulatory requirements, such as ensuring the ongoing airworthiness of the aircraft, operating within weather requirements and ensuring crew have the appropriate operating experience, including holding a Commercial Pilot License (CPL). While the regulations set the minimum standards, Part 135 type operations do not have the same degree of systems and procedures as larger airlines. In addition, many pilot seeking to progress to the airlines must first build flight time and experience as a commercial pilot. Commercial pilots often fly single-pilot, in remote areas, with limited direct supervision, requiring a greater level of decision-making autonomy. These challenges can lead to pilots pushing their own and/or operational limits, as demonstrated by a heli-skiing accident, near mount Aspiring National Park, New Zealand (5).

With aviation accidents in general having a low likelihood of occurring, risk-taking behavior may appear to have little consequence at the time, and the behavior can become normalized (6). A pertinent example is the continuation of a VFR flight into IMC, which is widely regarded as a significant and continuing factor in many aviation accidents. Research conducted by the Australian Transport Safety Bureau (ATSB) (4) shows that although the dangers of flying VFR into IMC are well known, VFR pilots still fly into deteriorating weather and IMC.

A review of publicly available aviation accident / serious incident reports, over a 10-year period up until January 2019, associated with Part 135 type operations from both Australia and New Zealand, identified the following predominant risk-taking behaviors as contributing factors: not following the published procedures (7–9), pushing the aircraft limitations when unsafe to do so (5, 10, 11) and continuation of the flight into deteriorating conditions (12–15). With approximately 80% of aviation accidents associated, at least in part with human factors (16), it is thus important to further understand the types of risk-taking behavior and the influences that may impact on the safety of these operations.

## Influences on and Incentives For Risk-Taking

Compliance with regulations, rules and procedures is deemed to be associated with individuals' safety motivation, although just because an individual has a high safety motivation does not mean they will never engage in rule-breaking behaviors (17). Pilots may violate due to viewing a rule or procedure as deficient, or too complex. Other's seek excitement and in rare cases some may intend to cause harm or sabotage (18). As such, this may provide a solid basis to explain a pilot's motivation to undertake risk-taking behavior for better, for worse, or even perhaps for indifferent.

Careful consideration, however, also needs to be given to understanding how an individual's behavior is influenced by the socio-technical system within which they operate. These influences can include socio-cultural, socio-political, and socio-psychological factors (19). Madhavan and Lacson (20) in their review of the factors that influence incidences of VFR into IMC report that a pilot's decision to engage in risk-taking can be influenced by social and peer pressures, such as wanting to impress the passengers.

These social and peer pressures can become normalized as the “way things are done”, leading to group and cultural norms, where routine violation and risk-taking behavior are accepted. Attitudes, beliefs, and the perceptions of group norms and values are often associated with the term safety culture. Petitta, Probst (21) suggest that safety culture strongly influences the way individuals act and react to risk. Chen and Chen (22) also found that a pilot's positive perception of the organisation's safety management system and thus their positive perception of the organizational culture was shown to have a beneficial effect on the pilots' safety motivation. Thus, it is important to consider not just the individual-level but also the group- and organizational-level factors that may influence risk-taking behavior.

## Safety Motivation and SDT

As stated, accidents in general have a low likelihood of occurring and thus the individuals may consider there to be no undesired outcome from undertaking risk-taking behavior. With extrinsic motivation relying on the mechanism of obtaining a reward or avoiding an undesired outcome (23), the individual will likely lack the extrinsic motivation to modify the behavior. This can lead to a mindset of “what's wrong with this way of behaving”. Similarly, if rule compliance offers little autonomy or interest–the individual does not find the activity personally rewarding or interesting, having no intrinsic motivation, then full compliance will not be guaranteed (24).

SDT provides an understanding of the relationship between individuals' safety motivation and safety behaviors through a continuum of extrinsic to intrinsic motivations, which is also referred to as more controlled through to more autonomous (23). The theory suggests that intrinsic motivation and the internalization of extrinsic motivation are determined by the degree to which people can satisfy the three basic psychological needs for autonomy, competence and relatedness to the environment in which the activity takes place.

When individuals are motivated to work safety because they view the activity as consistent with their values and interests (25), the safety motivation is “autonomous”. By making an individual feel pressured to perform an activity and the individual feeling obligated to do it, the individual's safety motivation is “controlled” (25). Within the context of SDT, autonomous motivation is distinguished as self-determined and controlled motivation as non-self-determined (26). Given the high degree of decision-making autonomy required by Part 135 pilots, the relationship between the potential multi-level influences and the pilots self-determined or non-self-determined motivation, may highlight important considerations for future research.

## Scope of the Review

Alper and Karsh (27) in their systematic review of safety violations in general industry settings, identified a lack of systematic research on the concepts and causes of violation. This lack of systemic research on violation and risk-taking behavior is reflected in the aviation context. Although, violations such as intentional VFR flight into IMC and the failure to follow procedures are frequently associated with aviation accidents (16), limited systematic research has been conducted on the subject. In their efforts to understand the psychological factors affecting pilots' decisions to navigate into deteriorating weather, Madhavan and Lacson (20) identified a number of factors from a review of the existing experimental data. Social pressure was one factor considered to have the potential to affect pilot decision-making, however it was concluded that the degree to which social pressure influences pilot decision making requires further exploration (20).

Multilevel theory proposes that, rather than concentrating on each separate level of organization, group or individual, an integrated approach be taken (28). Appreciating the socio-technical system as a whole, and taking a “systems thinking” approach (29), the current systematic review of the literature will aid in determining how these multilevel influences and/or incentives relate to risk-taking behavior in general.

Understanding the types of risk-taking behavior and multilevel influences/incentives, could help facilitate mechanisms to increase the intrinsic motivation to conduct the desired behaviors, such as explored by Scott, Fleming (25).

Our goal was thus twofold, firstly to take stock of the existing literature to investigate the types of risk-taking behavior empirical research has focused on, and to compare that to the types of risk-taking behavior identified in aviation accident reports from Australia and New Zealand. Secondly, to thematically synthesize, analyse, evaluate and explain academic research regarding the factors that incentivise and/or influence pilots to engage in risk-taking behavior, and consider the potential relationship with safety motivation.

Accordingly, the following questions were posed:

- What types of risk-taking behavior are the focus of empirical research and how do these behaviors relate to risk-taking identified as contributing factors in Part 135 type operational incidents and accidents in Australia and New Zealand?
- What are the multilevel influences/incentives that lead pilots to engage in risk-taking behavior?

Our hypotheses were thus:

1) The risk-taking behavior of VFR flight into IMC will be of a focus in the empirical research due to its prevalence as a significant safety risk related to worldwide aviation accidents.
2) The types of multilevel influences/incentives that lead to pilots engaging in risk-taking (VFR flight into IMC) will show parallels with safety motivational constructs.

## Method

To provide a comprehensive synthesis and to minimize the chance of bias, the review followed the Preferred Reporting Items for Systematic Reviews and Meta-Analyses (PRISMA) guidelines (30). Although PRISMA generally focuses on the reporting of reviews evaluating randomized trials, it presents a methodological framework for structured literature reviews (30).

**Figure 1:**
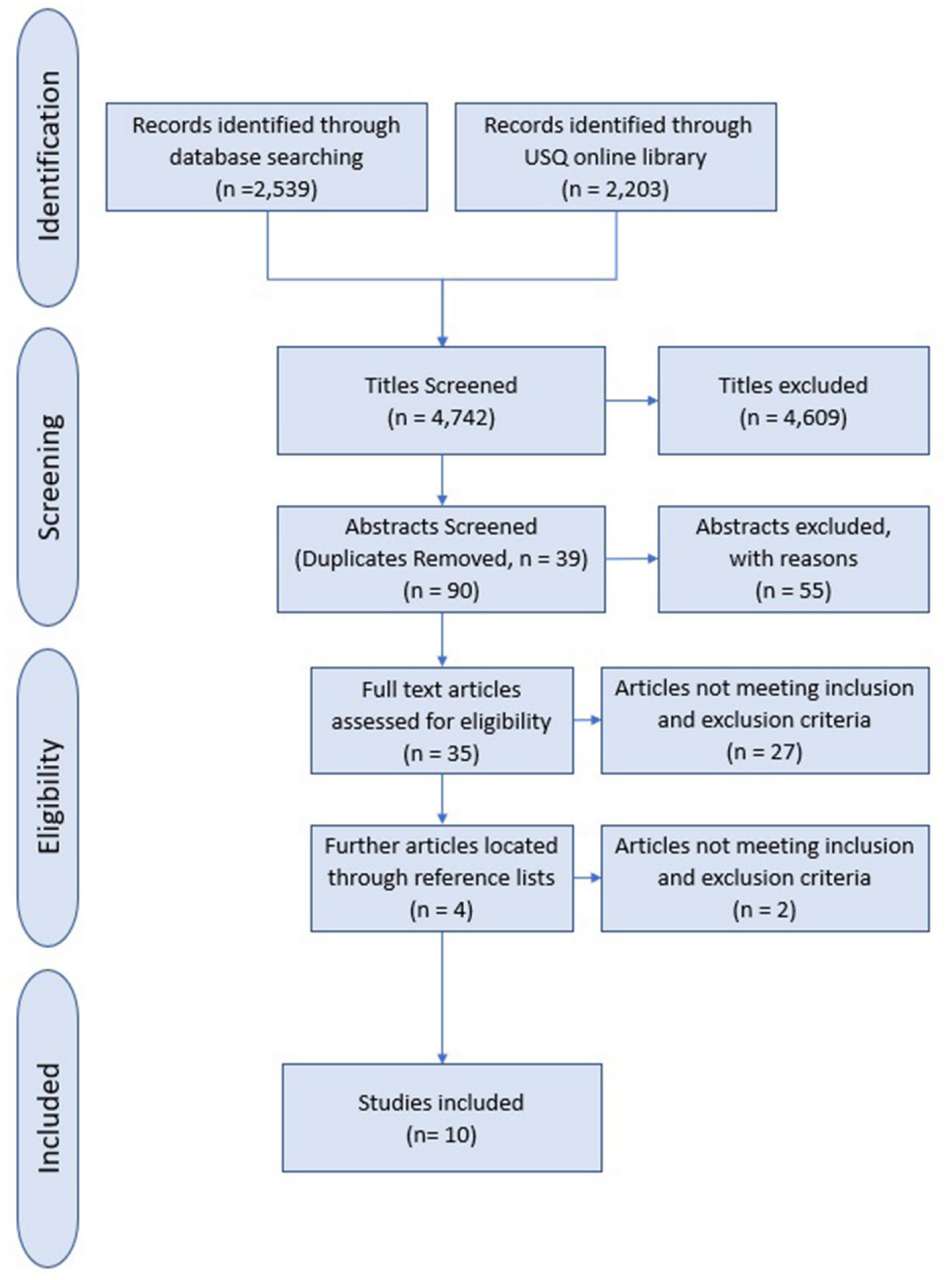
Flow diagram indicating the search procedure based on PRISMA guidelines.

The PRISMA guidelines set out 27 criteria for best practice in the design and conduct of systematic literature reviews and meta-analyses, with a particular emphasis on the consideration of sources of potential bias. Bias can occur across multiple areas within a review including how the eligibility criteria are determined, how information sources are used, and how potential bias in selected studies are evaluated (30). Figure 1 illustrates the selection process followed based on the PRIMSA flowchart template (31).

### Literature Search

The primary method for identifying relevant literature was a systematic search of electronic databases using a single Boolean search string, to identify only full text articles in English. To capture the broader context of risk-taking the following key terms were used for all searches (where “^*^” is the wildcard term used for truncation);

- “risk-taking” or “risk perception” or “risky decisions”
- “viola^*^” or “unsafe act^*^” or “non-compliance”,.
- “pilot” or “aircrew” or “crew”.

The following additional terms were added where necessary, to narrow the search results, using the AND NOT Boolean operator;

- “unmanned” or “military” or “army” or “navy” or “air-force” or “defense”
- “health” or “medic^*^” or “surgery.”

Searches were adjusted to best suit the search characteristics of each database. The academic databases searched were;

- EBSCOhost E Journal (PsycArticles and PsycINFO),
- ScienceDirect,
- Taylor and Francis Online,
- SAGE, and
- Springer.

The secondary method for identifying relevant articles was a search of the University of Southern Queensland (USQ) online library using two single Boolean search strings. The citations of the articles that met the inclusion criteria were also reviewed.

### Inclusion and Exclusion Criteria

The research team reached consensus on the inclusion and exclusion criteria before the review was conducted. The inclusion and exclusion criteria for the articles are shown in Table 1.

**Table 1:** Inclusion and exclusion criteria.

**Inclusion criteria**	**Exclusion criteria**
A focus on risk-taking; intentionally deviating from minimum standards (violation) or knowingly pushing ones' own or the aircraft safety limitations	The subject matter related to any aircraft that is not eligible for operation under Part 135 type operations (helicopter or small airplanes only)^2^
A focus on identifying variables that influence/predict/incentivise violation or risk-taking relating to aviation safety standards or regulations	The subject matter related to single engine instrument flight rules passenger operations^3^
The participants were commercial pilots (held a Commercial pilot license) or were operating under the requirements of Part 135 (or equivalent)^4^	The participants did not include commercial pilots or were solely: non-pilots/student or trainee pilots/airline transport pilots/military pilots
The article was available online	The subject matter did not relate to aviation
The article was written in the English Language	None of the inclusion criteria are met

### Inclusion of Studies

At the identification stage 2,539 records were returned via academic database searches and another 2,203 records were sourced from the USQ online library (see Figure 1). These records consisted of peer reviewed articles and conference proceedings published prior to August 18, 2019. During the initial screening phase, the record titles were assessed for adherence to the inclusion and exclusion criteria. Through this process 4,609 were excluded for two predominate reasons: The record did not relate to the “act” of intentional risk-taking/violation and/or the title indicated the record was obviously of a non-aviation context. An example being the article titled “Surgeons' intraoperative decision making and risk management” (32).

After duplicates were removed (*n* = 39), 90 records were then screened against the inclusion and exclusion criteria through reading the title and abstract. 55 articles were excluded at this stage and the full text for the remaining 35 articles was retrieved. The 55 records were excluded for one of three reasons:

- The study was not aviation related, an example being the assessment of risk-management and rule-compliance in hazardous industries, conducted by Hopkins (33).
- The participant group did not include commercial pilots, for example the study of pilot procedure-following behavior conducted by Landry and Jacko (34), which employed 12 male airline pilots.
- The study related to more broad non-intentional actions, or risk factors relating to accidents. An example being the study of human risk factors associated with pilots in runway excursions (35).

At the next stage, the primary author reviewed the full text and also the records within the reference lists of each of the 35 articles retained. Review of the reference lists identified four articles that had not be identified *via* database or library searches and appeared to meet the inclusion criteria based on their title. The full text for these articles were also retrieved and reviewed. Assessing these articles against the inclusion and exclusion criteria lead to the inclusion of two articles and the exclusion of two articles due to one of the three reasons stated above.

With the specific aim of the review being to assess and evaluate the risk-taking behavior of pilots operating under Part 135 type operations, it was considered and agreed on by the research team to limit studies to only those engaging actual pilots and specifically Part 135 eligible pilots in the final selection. Consequently, the primary author assessed the 37 articles for study design and the inclusion of commercial pilots in the participant group, which resulted in the exclusion of 16 articles.

### Final Selection

The full text of each of the remaining 21 articles were then reviewed by the research team and each article was independently assessed and rated against the inclusion and exclusion criteria. An article was assessed to have met the inclusion criteria if the reviewer judged the article to meet at least one of the first two inclusion criteria and all of the other inclusion criteria. In all but one case, all articles met all five inclusion criteria. The one exception was where the focus of the study was on decision making rather than risk-taking, but the decision-making related to violation of safety rules and the influences/incentives on those violations (36). An article was assessed to have met the exclusion criteria if the reviewer judged the article to not meet at least four inclusion criteria, or it met one or more of the exclusion criteria.

The research team consisted of three reviewers' the primary and secondary authors and (the third author acting as) an independent reviewer. Based on the 21 pairs of ratings for all records associated with the primary and secondary authors' reviews, an interrater reliability coefficient of Cohen's Kappa was initially calculated. The Cohen's Kappa was estimated at 0.71, which indicated substantial agreement (37). Initially it was agreed that 10 articles met the inclusion criteria and eight articles met the exclusion criteria. Of the 21 articles there were only three cases of non-agreement. In these three cases of non-agreement, the disagreement was resolved based on closer review of the participant group/study design, and in all three cases it was agreed that no commercial pilots were engaged as part of the study. This resulted in the exclusion of all three articles.

The independent reviewer agreed with the judgement of all 10 articles determined to meet the inclusion criteria. Of the 11 articles judged to have met the exclusion criteria the independent reviewer disagreed on six cases. In these six cases of non-agreement, the reviewers together agreed to exclude the studies after discussing their interpretations of the first and second inclusion criteria, reaching consensus.

The studies were exclude based on:

- three of the studies not specifically engaging commercial pilots, and
- three of the studies having neither a focus on risk-taking nor a focus on the factors that may influence/incentivise the behavior.

This resulted in a total of 10 final articles moving forward for quality appraisal.

### Quality Appraisal

There are various quality checklists that can be used for systematic reviews, including the Cochran Collaboration Risk of Bias Tool (38), and the Joanna Briggs Institute (JBI) Critical Appraisal Tools (39).

For this review, quality appraisals were undertaken for each study, to assess the methodological quality and potential bias in the design, conduct and analysis, utilizing the JBI Critical Appraisal Tools (40). The studies were assessed against each criteria, on the basis of whether the standard set was either met/or not met and assigned a value of either 1 or 0 respectively. Assessments were only compared once initial appraisal of all 10 articles was completed by both reviewers. The reviewers agreed that a score of 0.8 or above was to be considered strong, scores <0.8 and above 0.6 were considered to be moderate, and scores <0.6 were to be considered weak.

Studies that did not receive an overall quality rating of strong were typically weaker because of selection bias and a less effective control of confounding variables. Some discrepancy was observed in the independent overall quality rating assigned to each record, resulting in a Cohen's Kappa of 0.43, which indicates a fair to good agreement. Where a lack of consensus was found, a discussion was held between the reviewers and a final overall appraisal score was agreed.

## Results

To assist with analyses and thematic syntheses of the findings across the articles the data has been presented in four tables. In Table 2, articles are grouped by quantitative studies (the first seven articles in Table 2) and qualitative studies (the remaining articles in Table 2). In summary Table 2 presents information on authors and year, study design and sample characteristics, measures used, the type of risk-taking assessed, key findings, quality assessment, limitations, and directions for future research suggested by each article.

**Table 2:** Summary of the 10 articles that met the inclusion criteria.

**References**	**Study design/sample**	**Measures**	**Quality assessment**	**Type of risk-taking**	**Key findings**	**Limitations**	**Directions for future research**
Wigginset al. (41)	Cross-sectional, survey/251 qualified pilots 30.3% (76) CPL, 69.7% (175) PPL 117 instrument rated 62 multi-engine rated	Risk perception test, and questions seeking: (a) pilots' minimum weather-related criteria for flight; and (b) the frequency with which pilots had been involved in hazardous events	1.0 - Rated strong	Visual flight into Instrument Meteorological Conditions (IMC)	(*p =* 0.01) Pilots who indicated that they did not see the deteriorating weather conditions tended to be involved in a significantly greater frequency of hazardous events than pilots who described the deterioration in the weather conditions as gradual or who did not realize the severity of the change (*p* = 0.03) Pilots who deliberately entered IMC indicated that they had experienced similar conditions more frequently than those pilots whose transition into IMC was inadvertent – no difference between groups, based on possession of an instrument rating (*p* = 0.15) (*p* = 0.03) Pilots who held an instrument rating and entered IMC deliberately, perceive a relatively lower level of generalized risk in comparison to those pilots who entered IMC deliberately, but did not hold an instrument rating (*p* = 0.04) Pilots who entered instrument conditions inadvertently, reported greater levels of anxiety, than those pilots who entered instrument conditions deliberately	Cross-sectional design, correlation not causation.. Data collected retrospectively	The development and evaluation of novel approaches to weather dissemination, display and training
Wiggins et al. (42)	Cross-sectional/ Phase one, feature identification /association task: 57 qualified pilots (55 male, 2 female) Aged 18 to 70 yrs. 60% (35) PPL 18.2% (10) CPL 21.8% (12) ATPL Phase two, flight simulation task: 20 VFR pilots (all male) subset of phase one	Phase one: Cue-based performance was assessed using a modified version of the EXPERTise SJT (43) Phase two: Simulation assessment of decision to continue or deviate from the planned flight.	0.88–Rated Strong	Visual flight into deteriorating weather conditions	(*p =* 0.022) Pilots whose performance was more consistent with greater levels of cue utilization recorded relatively dichotomous responses either to conduct or not conduct the flight. (*p =* 0.025) 77% (7) Pilots whose performance was consistent with higher level of cue utilization continued the flight compared to 27% (3) whose performance was consistent with lower levels. No significant relationship between pilots' risk perception scores on the basis of whether they continued or diverted from the planned flight. No differences were evident between pilots who diverted from the planned route and those who continued in terms of the total number of flight hours accumulated, and number of hours accumulated in the preceding 90 days.	Cross-sectional design, correlation not causation	To adopt a similar approach across other forms of situation assessment under uncertainty, including the interpretation of weather radar displays and the analysis of the decision height for instrument approaches to landing
Hunteret al. (44)	Cross-sectional, survey/ 364 Participants Aged 16 to 76 yrs. 59% (215) PPL 25% (91) CPL 16% (58) APTL/Other	Risk perception scale, pilot judgement scale and questions seeking information on the weather-related event, circumstances, and reasons for involvement in the event	0.81–Rated Strong	Visual flight into adverse weather conditions	(*p =* 0.01) The in-weather group had the largest number of hazardous events. (*p =* 0.01) When asked whether they had previously flown in adverse weather conditions or in IMC, significantly more of the near-weather group 77% than the in-weather group (61%) indicated they had done so. (*p =* 0.02) In-weather group had the lowest scores on pilot judgement scale. (*p =* 0.01) Groups significantly differed with respect to possession of an airplane instrument rating. This rating was held by 43%, 59% and 41% of the in-weather, near-weather and no-weather groups, respectively. No difference between groups on their risk perception	participants represent a sample of convenience exploratory study, no experiment-wide error correction attempted.	The degree of similarity between the pilots in this study and the pilots involved in weather-related accidents should be investigated further
Pauley et al. (45)	Cross-sectional/ Phase one, sorting task: 23 qualified pilots (20 male, 3 female) Age 17 to 58 yrs. 52% (12) PPL 44% (10) CPL 4% (1) ATPL Phase two, sorting task: 32 qualified pilots (30 male, 2 female) Age 18-65 yrs. 50% (16) PPL 44% (14) CPL 6% (2) ATPL/Other	Phase One: Hazardous Events Scale survey. Implicit associations between depictions of VMC and IMC weather conditions and sets of words meaning risky and safe. Phase Two: Anxiety IAT and Risky IAT	0.75–Rated Moderate	Visual flight into adverse weather conditions	(*p =* 0.17) The low-involvement group (n=9) had a significantly stronger IAT effect than the high-involvement group (n =14). The weaker the pilots implicit association between adverse weather and risk, the more hazardous events in which the pilot had been involved, such as flying in bad weather (*p =* 0.035) Significant positive relationship between the frequency with which pilots had been involved in weather-related incidents and the strength of their association between IMC and feeling afraid and between VMC and feeling unafraid, as compared to the reverse. (*p =* 0.006) Significant relationship between the number of hazardous events in which the participant had been involved and the D (difference) score for the anxiety IAT. *The positive relationship suggested that the greater the number of hazardous events in which the participant had been involved, the higher the D score. Participants with a higher IAT score had weaker associations between IMC and feeling afraid and between VMC and feeling unafraid, as compared to the reverse*. (*p =* 0.013) Significant correlation between the D scores for the risky and anxiety IATs. Participants who more strongly associated IMC conditions with risk also had stronger implicit associations between IMC and anxiety.	Cross-sectional design, correlation not causation	The relationship between implicit anxiety and Weather-related decision-making during a simulated flight. Further exploration / comparison between men's and women's implicit attitudes, might be an important, and hitherto unexplored, factor in explaining these differences
Pauley et al. (46)	Cross-sectional/ Phase one (scenario development): 4 Pilots (3 male, 1 female) Aged 21 to 31 yrs. 3 CPL, 1 ATPL Phase two, decision task-based: 27 qualified pilots (24 male, 3 female) Aged 21 to 54 yrs. 19% (5) PPL, 81% (22) CPL Phase three, flight simulation task: 32 qualified pilots, (30 male, 2 female) Aged 18 to 65 yrs. 50% (16) PPL, 44% (14) CPL, 6% (2) ATPL/Other	Study One: Participants ranked scenarios by level of opportunity or threat presented. Study Two: 6-point Likert scale from definitely no to definitely yes - wiliness to undertake flight scenario. Flight Simulator Study: Decision to divert or turn aircraft back, during a lowering cloud base scenario	0.75 - Rated Moderate	Visual flight into adverse weather conditions	(*p* < 0.01) In all cases, the pilot's responses were significantly influenced by the level of threat in the situation. Participants who continued tended to be less influenced by threat compared to the participants who diverted, suggesting that the continuers were less risk averse. (*p =* 0.034) When threat was weather and the opportunity was income earned, two pilots showed risk tolerant tendencies. The two risk tolerant pilots had been involved in significantly more hazardous events compared to the risk adverse pilots. No significant difference in the age or experience level between the two groups of pilots. This suggests that the difference in prior exposure to hazardous events between the risk tolerant and risk adverse pilots is not mediated by flight experience Those who continued tended to be less influenced by threat compared to the participants who diverted, suggesting that the continuers were less risk averse than the diverters	Cross-sectional design, correlation not causation The type of decision-making measured differed between studies (decision to conduct/ decision to continue).	Explore whether training pilots to attend to the threat of loss and to ignore the opportunity for gain will improve decision-making?
O'Hare and Smitheram (47)	Quasi-Experimental, decision task-based/ 24 pilots (all Male) Aged 18 to 46 yrs. 33% (8) Student pilots 54% (13) PPL 13% (3) CPL	Continue/discontinue decision based on scenario of inflight weather, manipulation of the framing of the scenario for losses/gains.	0.72 - Rated Moderate	Visual flight into adverse weather conditions	(p <0.05) Pilots in the loss frame were significantly more likely to elect to continue with the flight than participants in the gain frame. Loss frame; a choice between the acceptance of a certain loss or risking further loss. Loss being the time and money invested so far in the flight.	Participants recruited from aero clubs (only 3 held CPL) may not be representative of the wider commercial pilot population	Augment the decision makers natural strategies with simple techniques derived from behavioral decision theory
Pauley and O'Hare (48)	Cross-Sectional/ Phase one (scenario development): 5 pilots Phase two, decision task-based: 27 pilots (inc. student pilots, instructors, and tourist flight operators)	Phase one: 6-point Likert scale used to determine high, medium, low level of opportunity or threat. Phase two: 6-point Likert scale - likelihood that the participant would take off on each flight	0.56 Rated Weak	Various scenarios relating to flights involving threats - including flying in adverse weather	(*p =* 0.04) Significant relationship between the overall influence of threat and the number of hazardous events experienced – This positive correlation suggests that the more incidents that the pilots were involved in, the smaller the influence of threat on the decision to take off. This implies that the participants who were less risk averse were involved in more hazardous incidents. (*p =* 0.02) The risk tolerant pilots had been involved in significantly more hazardous events compared to the risk averse participants. – Risk tolerance was related to risk-taking behavior.	Cross-sectional design, correlation not causation	Assess the relationship between risk tolerance and risk taking in a simulated flight
Michalski and Bearman (36)	Qualitative/Semi-structured interviews 12 Pilots (9 Male, 3 Female), Aged 24 to 63 yrs. *although not specified it is considered likely that all pilots held at least a CPL, due to the interview questions relating to jobs held in the outback–requiring a pilot to hold commercial license	Factors affected the decision making of participants flying in the Australian Outback	0.90 - Rated Strong	Not following rules, regulations or procedures. E.g., flying low level	Thematic analysis identified a number of challenges that were classified according to the broad categories of organizational, social and personal factors Organizational factors identified were: organizational culture, time-pressure and fatigue Social factors Identified were: social culture and customer pressure Personal factors were career ambition	Small sample size Participants may not have been willing to disclose stories of situations in which they made an error of judgment	Determine how widely generalizable the pressures identified in this study are with regards to other remote locations
Bearmanet al. (49)	Qualitative/Semi-structured interviews - critical decision method 24 Pilots (all Male) Aged 31 to 69 yrs. 87.5% (21) held CPL/ATPL 12.5% (3) held PPL	Situational pressures on decision-making associated with weather-related incidents that had challenged the participants' skills as a pilot	0.80 - Rated Strong	Various scenarios relating to flights involving threats/risks - including visual flight into adverse weather.	Situations that motivated pilots toward unsafe behavior were labeled as “goal seduction”. E.g. feeling pressured to reach their destination by monetary factors or 'being in 'search and rescue' mode - “when you think that you're going to save somebody, you'll push things.” Situations that motivated pilots away from safe behavior were labeled as “situation aversion”. E.g. not wanting to land where there was a lack of basic facilities	Data collected retrospectively Small Sample Size	Determine the extent to which pilots flying normal operations are subject to the influence of strong situations
Paletz et al. (50)	Qualitative/ Semi-structured interviews - critical incident technique 24 Pilots (all Male) Age 31 to 69 yrs. 87.5% (21) held CPL/ATPL 12.5% (3) held PPL	social pressures associated with situation involving weather when participants were pilot in command and found their skills challenged	0.75 - Rated Moderate	Visual flight into adverse weather conditions	Of the 24 pilots, 16 described pressures that were coded specifically as social psychological (see Table 1). Seven interviews contained informational social influence, three contained foot-in-the-door, five contained normalization of deviance, and five contained self-motives	Small Sample Size Other mechanisms could also be at work	Further evolution of Human Factors Analysis and Classification System by taking advantage of social psychological theories

*PPL, Private Pilot License; CPL, Commercial Pilot License; ATPL, Airline Transport Pilot License*.

Table 3 presents a summary of information from the seven quantitative studies organized across potential associations–namely the types of influences that may show association with a deteriorating weather / IMC encounter. In addition, Table 3 present a subset of potential influences that may be associated with previous involvement in hazardous events, (where a deteriorating weather / IMC encounter would be considered a hazardous event).

**Table 3:** Influences on risk-taking–quantitative studies.

**Deteriorating weather / Instrument Meteorological Conditions (IMC) encounter**	**Association**	**No association**
Previous involvement in hazardous events	(*p =* 0.01) Wiggins et al., (41)	
	(*p =* 0.01) Hunter et al., (44)	
Prior experience of similar conditions	(*p =* 0.03) Wiggins et al., (41)	
	(*p =* 0.01) Hunter et al., (44)	
Perception of risk / risk tolerance / risk aversion	(*p =* 0.03) Wiggins et al., (41)	Wiggins et al., (42)
	(*p =* 0.17) Pauley et al., (45)	Hunter et al., (44)
	(*p <* 0.01) Pauley et al., (46)	
Judgement	(*p =* 0.02) Hunter et al., (44)	
	(*p =* 0.022) Wiggins et al., (42)	
	(*p =* 0.025) Wiggins et al., (42) (*p <* 0.05) O'Hare and Smitheram, (47)	
Perceived anxiety / fear	(*p =* 0.04) Wiggins et al., (41)	
**Previous involvement in hazardous events**.		
Perception of risk / risk tolerance / aversion	(*p =* 0.034) Pauley et al., (46)	
	(*p =* 0.04) Pauley and O'Hare, (48)	
	(*p =* 0.02) Pauley and O'Hare, (48)	
Perceived anxiety / fear	(*p =* 0.006) Pauley et al., (45)	

Table 4 presents the factors influencing pilot's decision making identified from the three qualitative studies, organized by organizational, social and personal factors. For each category examples are given from the qualitative studies to help explain the concepts of interest.

**Table 4:** Influences on risk-taking–qualitative studies.

**Organizational factors**	
Normalization of deviance	Michalski and Bearman, (36) “*One comment made by a pilot to justify committing violations demonstrates just how normal these attitudes were: ‘you've got to turn a blind eye to some of it otherwise you're not going to be able to work and it's not going to be a productive business.”'*
	Paletz et al., (50) “*Pilots describe becoming used to the risk of flying in bad weather and the dangers of complacency. When the same risky behavior led to no negative consequences, the pilots kept performing the same behaviors during the course of several days or flights. ‘It could stay that way for weeks and weeks, foggy and wet, rainy, and you get out there and fly and you get accustomed to it'.”*
Direct report influence–foot-in-the-door	Bearman et al., (49) ”*'He went to have a look-see and that was the end of that. Didn't even get in the pass – got in the pass but never made it around the corner.”'*
	Paletz et al., (50) “*'[A manager would say] ‘why don't you go take a look? See what it looks like. It's legal to leave – go look.' You get out there and generally you don't come back. You're already out there […] So you just keep skulking and skulking under this bad weather a little bit more, until all of a sudden you're in over your head […]”'*
Organizational pressure–time / financial	Bearman et al., (49) “*In some cases pilots were only paid for completing a flight as scheduled. This exerted a powerful incentive to reach their destination, even if doing so was not the safety option.”* “*A pilot had a fatal accident because he only had one chance to make a trip: ‘it was windy and Gusty and just not very good to go in there. It was one of those let's go take a look at it cause this will be the only chance we'll get to do the trip… He was supposed to leave the day before and the weather wasn't good.”'*
	Michalski and Bearman, (36) “*'There's 400 other guys on file with resumes and I can have any one of them here tomorrow. Do you want the job? Make me money.”'* “*'When you get back from a flight, you'd have about 40 min to check the weather, do a flight plan, refuel the aircraft, have your lunch, and get all these things done. You just don't have the time to do all that stuff comfortably, so you'd go have lunch and you'd make it a 40 min lunch break and you jump back into the airplane with the same flight plan, and the same weather that you left with in the morning.”'*
**Social factors**	
Being accepted as part of the group / informational social factor	Michalski and Bearman, (36) “*pilots often gave into social pressures because they wanted to fit in and be accepted by their peers”*.
	Paletz et al., (50) “*'He went through the pass and he got through it just fine, but the 10 it took me to get to where he was, the pass had closed.' The assumption of safety based on observation of others' flying may be incorrect because weather can change rapidly, the other pilots could be taking great risks, and/or the other pilots may be more experienced, equipped, or knowledgeable then the observer.”*
Perceived customer pressure	Michalski and Bearman, (36) “*Pilot attempted to land at the destination site in stormy conditions so she could pick up a customer who needed to attend a funeral. Pilot faced requests to fly lower than the regulations allowed and to go to other destinations than those specified in the original booking. Several pilots admitted to giving into these pressures and conforming to what was requested, often because the pilot was concerned that the customer would complain to their boss.”* Paletz et al., (50)
	“*'it takes a lot to look at these four people and go well, I know you see the lake [his passengers wanted to visit] but I don't want to go in there because its dangerous.' Feeling pressured to avoid social disapproval and failure (e.g. reluctance to disappoint passengers). In such cases, the passengers did not necessarily express disappointment; the pilot was simply aware of the passengers' desire and wanted to fulfill them.”*
**Personal factors**	
Personal benefit	Bearman et al., (49) “*'I said well, you know, it's pretty bad and I went. I literally went through indefinite ceiling, half mile, quarter mile junk for the better part of the pass… but that was a stupid decision based on wanting to get home.”'*
	Michalski and Bearman, (36) “*'because night hours are really hard to come by I decided to leave even earlier so the whole flight would be at night. So it meant getting up at 1 am so I didn't get much sleep and I was in a (Cessna) 210 with no weather radar and I basically just blasted off without thinking too much about it and there were just thunderstorms everywhere and lightning flashes, but the problem at night is that you can't tell how close they are. So I was just kind of flying blindly in the direction I needed to go to. I ended up flying through a bit of weather at some stage, which was rough.”'*
Reluctance to admit defeat / incur personal inconvenience	Bearman et al., (49) “*Reluctance to land in remote areas–because of the discomfort/inconvenience. Decision making was influenced by not wanting to land where there was a lack of basic human facilities. No sleeping accommodation for those who might get stuck in bad weather, forcing them to sleep in the aircraft, or ‘maybe on the floor of a shack somewhere'. The influences need not be large or important to others. Personal inconvenience of not having running water or telephone communications may be enough to subtly erode safety for particular people circumstances.”*
	Paletz et al., (34) “*Reluctance to face social disapproval was at times not very subtle, such as in situations in which the pilot might ‘lose face' or admit defeat in front of his or her peers. ‘Ego plays a big role in pushing a pilot to do something that, you know, he doesn't want to come back and say I couldn't make it […], three other pilots made it; what's wrong with you?”'*

Table 5 presents all of the associations identified across all studies, both quantitative and qualitative organized by two overarching themes developed through the thematic synthesis. To reduce the potential for biases when coding the data and developing the overarching themes, the Computer Assisted Qualitative Data Analysis Software NVivo (51) was utilized.

**Table 5:** Overarching influence themes.

**Continuation influence**		
*Personal factors*		
	Judgement	Hunter et al., (44)
		Wiggins et al., (41)
		O'Hare and Smitheram, (47)
	Personal benefit	Bearman et al., (49)
		Michalski and Bearman, (36)
	Reluctance to admit defeat / incur personal inconvenience	Bearman et al., (49)
		Paletz et al., (50)
*Social factors*		
	Perceived customer pressure	Michalski and Bearman, (36)
		Paletz et al., (50)
*Organizational factors*		
	Direct report influence – foot-in-the-door	Bearman et al., (49)
		Paletz et al., (50)
	Organizational pressure - time / financial	Bearman et al., (49)
		Michalski and Bearman, (36)
**Acceptance of risk / normalization of deviance**		
*Personal factors*		
	Perception of risk / risk tolerance / risk aversion	Wiggins et al., (41)
		Pauley et al., (45)
		Bearman et al., (49)
		Pauley and O'Hare, (45)
	Judgement	Hunter et al., (44)
		Wiggins et al., (41)
		O'Hare and Smitheram, (47)
	Previous involvement in hazardous events / Prior experience of similar conditions	Wiggins et al., (41)
		Hunter et al., (44)
	Perceived anxiety / fear	Wiggins et al., (41)
		Pauley et al., (45)
	Reluctance to admit defeat / incur personal inconvenience	Bearman et al., (49)
		Michalski and Bearman, (36)
		Paletz et al., (50)
*Social factors*		
	Being accepted as part of the group / informational social factor	Michalski and Bearman, (36)
		Paletz et al., (50)
*Organizational factors*		
	Direct report influence–foot-in-the-door	Bearman et al., (49)
		Paletz et al., (50)
	Normalization of deviance	Michalski and Bearman, (36)
		Paletz et al., (50)

### Analysis and Synthesis

Of the 10 records eligible for inclusion (36, 41, 42, 44–50) the seven quantitative studies yielded data from 837 individual pilots, with the smallest sample of 24 and the largest of 364 (M = 93.00, SD = 125.26). The total excluded nine pilots involved in scenario development for two studies (refer Table 2). Of the total participant group (*n* = 837), 29% of pilots held a CPL, while the majority (60%) held a Private Pilot License (PPL). The three qualitative studies yielded data from 60 pilots, with the smallest sample of 12 and the largest of 24 (M = 20, SD = 9.93). Approximately 90% of the total participant group (*n* = 60) held at least a CPL and/or an airline transport pilots license (ATPL).

Participants were recruited through various means including aviation websites, local aeroclubs and email distribution lists. Data were collected from across five regions: New Zealand (41, 44–47), Australia (36, 44–46), North America and Norway (41, 44) and Alaska (49, 50).

One of the quantitative studies was of a quasi-experimental design, while all other studies were of a cross-sectional design. Six out of the seven quantitative studies were rated as moderate or strong quality, with the remaining study (48) rated as weak quality. This study was one of the cross-sectional designs and was rated poor due to lack of clearly stated strategies to deal with confounding factors and ill-defined participant inclusion/exclusion criteria. All three qualitative studies consisted of semi-structured interview design, predominantly employing the critical incident method/technique (52). Two studies were rated strong, and the other rated moderate (50), due to the potential influence from the researcher on the research not being specifically addressed in the article and there being no clear statement on the ethical process undertaken.

### Types of Risk-Taking Identified

Table 2 shows that visual flight into deteriorating weather conditions, commonly referred to as VFR flight into IMC, was the primary phenomena of interest in six of the seven quantitative studies (41, 42, 44–47). The seventh study explored VFR flight into IMC as part of a number of scenarios (48). When asked to relate situations in which the pilots had found themselves when flying commercial operations, and the decision-making associated with these situations, VFR flight into IMC was the primary focus of two of the qualitative studies (49, 50) (refer Table 2). Considering the third qualitative study (36), VFR flight into IMC was raised as an example of when social and personal factors had influenced decision making (refer Table 4).

Considering the primary phenomena of interest in the majority of studies was that of VFR flight into IMC, the rest this review predominantly focuses on the potential incentives/influences that may lead pilots to engage in the risk-taking activity of continuing a VFR flight into deteriorating weather / IMC.

### Incentives and Influences on Risk-Taking

#### Quantitative Studies

The top half of Table 3 presents the possible associations related to involvement in a deteriorating weather / IMC encounter, identified across the quantitative studies. Six studies from 1995 to 2014 are listed, with a total of 810 participants included in this group, and 32.7% of this total participant group representing pilots who held a CPL. The participants in this group were from New Zealand, Australia, North America, and Norway. Five potential associations were investigated across the studies, these were:

- previous involvement in hazardous events (41, 44),
- prior experience of similar conditions (41, 44),
- perception of risk / risk tolerance / risk aversion (41, 42, 44–46),
- judgement (41, 42, 44, 47), and
- perceived anxiety/fear (41).

Two studies indicated an association between frequency or number of previous hazardous events the pilot has been involved in and the likelihood of the pilot having a deteriorating weather / IMC encounter (41, 44). Variations to the Hazardous Event Scale (HES) (53) were used to measure the frequency or number of hazardous events the pilots had previously experienced. Hazardous events related to the HES are occurrences such as low fuel states, geographical disorientation, and diversion due to deteriorating weather (54). The same two studies again employing modified HES also suggested an association between a deteriorating weather / IMC encounter and the pilots prior experienced of similar conditions (adverse weather / IMC) (41, 44).

Three studies identified an association between the pilot's risk perception or how risk tolerant / risk averse the pilot was to the risk of a deteriorating weather / IMC encounter and the likelihood of them entering the conditions (41, 45, 46). Two studies utilized a modified Risk Perception Scale (55) to measure the pilot's perceived level of risk tolerance and/or aversion. The third study employed the Implicit Association Test to measure implicit association between risk and IMC. Wiggins et al. (42) however, found no significant relationships between a pilots' risk perception and whether they continued or diverted from a planned flight, due to deteriorating whether conditions. When exploring the situational and personal characteristics associated with adverse weather encounters by pilots, no difference was found between the pilot's risk perception and whether or not the pilot had experienced a weather encounter (44).

An association between pilot judgment and a deteriorating weather / IMC encounter was indicated by three studies (42, 44, 47). Hunter et al. (44) employed an abbreviated version of the Pilot Judgement Scale (56) to measure pilot judgement. Wiggins et al. (42), investigated a pre-flight and in-flight weather decision making scenario using a variation of the EXPERTise programme (43) to measure the pilots' cue utilization. O'Hare and Smitheram (47) used the concept of framing manipulation to determine the potential influence on the pilot's decision making.

The bottom half of Table 3 expands on the concept of previous involvement in hazardous events identified across the quantitative studies and the possible related associations. The Hazardous Event Scale utilized by a number of studies describes a VFR flight into IMC as a hazardous event. Thus, the potential associations related to previous involvement in hazardous events, may also show similarities to those related to a deteriorating weather / IMC encounter. Three separate studies are listed with a total of 141 participants included in this group (45, 46, 48). One study did not specify the number of pilots holding a CPL in the participant group and thus of the total participant group (n = 141) at lease 60 (42.5%) held a CPL. Two potential associations were identified which showed similarities to the associations identified in relation to a deteriorating weather / IMC encounter, listed in column one. These were “perception of risk / risk tolerance / aversion” (46, 48) and “perceived anxiety/fear” (45). The studies were conducted from 2006 to 2008. The participants in the total sample were predominantly from Australia and New Zealand and all studies were cross-sectional design.

Two studies identified an association between the frequency of involvement in previous hazardous events and the pilot's risk perception or how risk tolerant/averse the pilot was (46, 48). A modified HES was employed by both studies.

Finally, one study suggested a significant relationship between the pilot perceived anxiety/fear and the frequency of which they had been involved in hazardous events (45). Measured via the anxiety Implicit Association Test (IAT) (57), the higher the IAT score, the weaker the associations between IMC and feeling afraid and between VMC and feeling unafraid, as compared to the reverse.

#### Qualitative Studies

Table 4 presents the possible associations identified across the qualitative studies which showed a potential association with continued VFR flight into IMC. As all three included qualitative studies are listed, the participant group was that of the total population (*n* = 60) with approximately 90% of the participant group holding at least a CPL and/or an ATPL. Three main themes emerged which are presented in Table 4; organizational factors, social factors and personal factors, and across the three themes seven potential associations were investigated, which are listed under column one. Both the organizational factors and personal factors themes were raised by all three studies; however, the social factors theme was only raised by two studies (36, 50).

Within the organizational factors theme, three predominant associations were identified with each association appearing to be common across at least two of the studies. The associations were: the normalization of deviance (36, 50), direct report influence (the foot-in-the-door technique being an example) (49, 50), and organizational pressure (such as time or financial pressures) (36, 49).

Two predominant associations were identified within the social factors theme. These were being accepted as part of the group / informational social factor and perceived customer pressure (36, 50).

Within the personal factors theme, two predominant associations were identified and again each appeared common across at least two of the studies. The associations were personal benefit (36, 49) and reluctance to admit defeat / incur personal inconvenience (49, 50).

### Safety Motivation

In total there are 12 distinct associations listed in Tables 3, 4. It is out of scope of this review to determine if each of these associations are indeed either an intrinsic or extrinsic motivator for the pilot's decision to engage in the risk-taking behavior. Each association could however, be considered to share characteristics of either an extrinsic motivation, the internalization of an extrinsic motivation, or an intrinsic motivation. Clear examples of extrinsic motivations are that of “direct report influence” (49, 50), “organizational pressure” (such as time or financial pressures) (36, 49) and “perceived customer pressure” (36, 50).

The associations of “previous involvement in hazardous events” (41, 44), “prior experience of similar conditions” (41, 44), “perception of risk / risk tolerance / risk aversion” (41, 42, 44–46) could be considered that of the internalization of the extrinsic motivation, under the construct of normalization of deviance.

## Discussion

Understanding the relationship between individuals' propensity to engage in risk-taking behavior during normal operations and their levels of safety motivation through a continuum of extrinsic to intrinsic motivation may have important implication for aviation safety. This understanding may also aid in the explanation of how multilevel influences affect individual's safety motivation. The aim of this review was to evaluate the types of risk-taking behavior investigated by empirical research compared to the types of risk-taking behavior identified in aviation accident reports from Australia and New Zealand. Secondly, to explore the potential multilevel influences and/or incentives that lead pilots to engage in risk-taking behavior, and consider their relationship to safety motivation via an SDT lens.

Our first hypothesis, that VFR flight into IMC would be identified as a focus of empirical research was supported. It is considered likely that the focus on VFR flight into IMC is due to the continued accidents associated with the phenomenon and the complex nature of the contributing factors. As stated by Wilson and Sloan (58), one of the leading causes of fatal aircraft accidents is the continuation of VFR flight into IMC. Our second hypothesis, that parallels could be drawn between the types of multilevel influences / incentives and individual's level of intrinsic and/or extrinsic safety motivation was also supported.

### Key Findings

#### The Types of Risk-Taking Behavior Pilots Engage in

Given the high proportion of academic focus on VFR flight into IMC, it is considered that this type of risk-taking behavior and the complex nature of the phenomena has needed and continues to deserve attention. This is supported by the real-world examples shown in the incident and accident reports from Australia and New Zealand. The continuation of flight into deteriorating conditions was considered one of the predominant risk-taking behaviors associated with aviation accidents related to Part 135 type operations in Australia and New Zealand. It should be noted however, that seven of the 10 studies were conducted by researchers from either an Australian or New Zealand University and data were collected from Australian or New Zealand pilot populations.

VFR flight into IMC was not the only type of risk taking of concern within the literature. Michalski and Bearman (36), identified that pilots also elected not to follow the rules, regulations or procedures in other aspects of their flying. An example of this was flying at an altitude below the minimum height rules for visual flight, of 500 feet (59).

#### The Multilevel Influences/Incentives That Lead Pilots to Engage in Risk-Taking Behavior

The reasons why pilots continue VFR flight into deteriorating weather conditions have been extensively investigated by academic institutes and numerous safety agencies. There are many factors which may influence and/or incentivise a pilot to engage in this type of risk-taking behavior, including situation assessment, risk perception, motivation, and decision framing (60).

From our systematic review of the academic literature, consensus was shown across the predominant types of influences/incentives. To best explain the multilevel constructs identified it is helpful to consider the associations presented in Tables 2, 3 in terms of two overarching themes, these being “continuation influence” and “acceptance of risk / normalization of deviance”. Table 5 presents both overarching themes and describes the related associations which can be considered in terms of personal, social and organizational influences and/or incentives.

##### Continuation Influence

**Personal Influence:** Four strong studies and two moderate studies indicated associations related to personal factors under the overarching “continuation influence” theme (36, 42, 44, 47, 49, 50). Exploring the situational and personal characteristics associated with adverse weather encounters, Hunter et al. (44), found that pilots in the “in-weather” encounter group (pilots who reported that, they, as pilot-in-command had entered IMC during VFR flight within the last 5 years) had the lowest scores as measured by the Pilot Judgement Scale (56). Pilots are constantly challenged to make the appropriate judgements and decisions in response to varying weather conditions, yet the outcomes of those decisions are not always favorable. Aviation accident and incident investigations have revealed that pilots frequently receive cues of deteriorating or hazardous weather conditions during the flight, yet continue (12, 15).

Investigating an in-flight decision-making scenario Wiggins, Azar (42) found that even with higher levels of cue utilization, pilots continued the flight despite deteriorating weather conditions.

The reason pilots elect to pursue a course of action even though indications are that an alternative course of action may be safer, is characteristic of plan continuation bias/error. This internally induced pressure or desire to get to the destination can be a powerful influence, with potentially fatal outcomes (15).

Wiggins et al. (42) however, suggest that “what presents as a plan-continuation error might be explained as a response to features, albeit inaccurate, that the rate of deterioration and the proximity of the destination are such that it supplants other features that might be associated with the safety of the aircraft”. The inappropriate or ineffective application of cues may be exacerbated by the gradual transition from minimum VFR conditions to IMC which can make the discrimination of weather conditions difficult. Given these circumstances pilots may be unable to detect the nuances that would lead them take a safer course of action, such as diverting. An example being where an experienced pilot of a Leonardo helicopters AW139, incorrectly assessed the in-flight conditions as visual meteorological conditions, entering cloud in the vicinity of Mount Baw Baw, Victoria, Australia, leading to a “caution terrain” alert activation (61).

There may also be practical reasons why pilots elect to continue, for example where the alternative is considered unacceptable and/or detrimental to individuals health and safety. Michalski and Bearman (36) identified that some pilots continued because the alternative involved putting themselves and others in a position/location with a lack of basic human facilities. This may include limited choice for accommodation, no running water or means of communication. When pilots were required to decide between the acceptance of a certain loss (wasting time, losing money, personal hardship, etc.) or risking potential further loss (the chance of having an accident) “the loss frame” vs. framing the decision as a gain, pilots in the loss frame were significantly more likely to elect to continue with the flight (47). Furthermore, when the decision not to conduct a flight means losing the potential to build “hard-to-come-by” hours, pilots may be more likely to undertake flights that could be considered unsafe (36).

**Social influence:** The role of societal pressure may also contribute to what has historically been called “get-there-it is” (58). One strong study and one moderate study indicated associations related to social factors (36, 50). In feeling pressured to avoid social disapproval and failure, particularly where the pilot was simply aware of the passengers' desire and didn't want to disappoint, this type of customer pressure can be considered a societal or social influence (50). The pressure to conform to what the customer wants can also be influenced by the concern that the customer would complain to the boss (36).

**Organizational influence: Two** strong studies and one moderate study indicated associations related to organizational factors (36, 49, 50). Organizational factors, such as an organisation's safety culture can significantly influence pilot's performance, both positively and negatively. A positive perception of the organizational culture can have a positive effect on pilots' safety motivation (22), however fear of reprisal, or of losing their job can equally influence a pilot's behavior in a negative way (36).

Some organizational influences however, are not as clear cut as “get the job done or we'll find someone else who will,” many can be considered indirect or “part of normal operations.” Influences such as time pressures, resource limitation and financial constraints/incentives. Having limited time to perform all the required tasks, can lead pilots to make trade-offs, either electing to omit doing a task, such as getting an updated weather briefing, or shortcutting procedures, such as rushing pre-flight checks, etc. (36). Bearman, Paletz (49), identified that for some pilots a powerful incentive to reach the destination, was the fact that some pilots were only paid for completing the flight as scheduled.

Other organizational influences are even more subtle. Examples of these influences may be social psychological pressures, such as the foot-in-the-door phenomenon or slippery slope fallacy. These phenomena are considered to be in effect where a person undertakes an action based on having already agreed to and undertaken a smaller task within a short timeframe (50). By directing a pilot to “go take a look”, the pilot has already essentially agreed to undertake the flight, the decision then becomes whether or not it is safe to continue.

##### Acceptance of Risk / Normalization of Deviance Influence

**Personal influence:** All studies indicated associations (with the seven quantitative studies showing significant associations) related to personal factors, under the overarching “acceptance of risk / normalization of deviance” theme. Given the context of the studies, this is not surprising, however, the relationships between the normalization of the risk and the multilevel constructs, which show similarities across the studies is significant.

Decision making under uncertainty involves the perception of risk, where risk can be defined as the likelihood of suffering a loss due to a hazard. In the case where the hazard is that of VFR flight into IMC, pilots may assess the situation accurately (i.e., detect the deteriorating weather), but they may not realize the risks involved in continuing with the flight (60). It is suggested that even when a hazard is diagnosed accurately, the individual's risk perception can be influenced by such factors as personal experience and ability (62). Pauley, O'Hare (46), Pauley, O'Hare (45), Wiggins, Hunter (41) indicate that the lower the risk perception and/or the less risk averse the pilots were, the more adverse weather events the pilots had encountered and the more likely the pilot was to deliberately enter the conditions. Pauley, O'Hare (46) also determined that pilots who continued into deteriorating weather conditions tended to be less influenced by threat compared to the participants who diverted, which suggested that the pilots who continued were less risk averse.

Conversely two strong studies, identified no significant relationship between pilots' risk perception scores and whether or not they entered/continued into deteriorating weather conditions (42, 44). This may be because, evidence suggests that most individuals possess a relatively accurate perception of the risks associated with specific activities, however, tend to perceive that the aggregate estimates of risk do not necessarily apply to them personally (63).

When faced with making a decision between a perceived “certain” risk (for example personal inconvenience/discomfort) vs. an uncertain risk in continuing (the chance of an accident occurring) pilots may consider the decision to continue as less risky (49). As such, personal inconvenience may be enough to subtly erode safety for particular pilots in particular circumstances, based on the pilot's perception of the risks they face and the trade-off they deem necessary. With decisions that involve a certain level of risk not always leading to unfavorable outcomes, the positive outcome associated with excessive risk-taking may potentially reinforce the behavior (64).

In the context of VFR flight into IMC, Hunter, Martinussen (44) suggest that IMC encounters may be relatively brief and otherwise uneventful, due to the gradually deteriorating weather conditions leading to the encounter. “The principal lesson that a pilot may take away from such a weather encounter is that he or she can enter IMC and survive unscathed” (44). When exploring the characteristics of pilots who report deliberate vs. inadvertent visual flight into IMC, Wiggins, Hunter (41) found no relationship evident between involvement in previous hazardous events in general and whether or not the transition into IMC was deliberate. However, pilots who deliberately entered IMC indicated that they had experienced similar conditions more frequently, compared to pilots who inadvertently entered IMC (41). Those pilots who entered instrument conditions inadvertently, also reported greater levels of anxiety, than those pilots who entered instrument conditions deliberately (41).

Investigating the implicit perceptions of risk and anxiety and pilot involvement in hazardous events, (45) found that qualified pilots all implicitly associated IMC with risk and VMC with safety. Pilots who more strongly associated IMC conditions with risk also had stronger implicit associations between IMC and anxiety. A significant positive relationship was however found which suggested that the greater the number of hazardous weather events in which the pilot had been involved, the less implicitly anxious he or she was to adverse weather.

These findings support the deduction that some pilots may tend to transition into deteriorating weather / IMC deliberately, on the basis that they a familiar with or have experienced relatively similar conditions, they perceive the transition into IMC as comparatively less risky, and experience lower levels of anxiety during the encounter (41).

**Social influence:** One strong and one moderate study showed associations related to social factors (36, 50). While one's desire to avoid perceived or actual social disapproval, to avoid looking “incompetent” by peers, to be accepted as part of the group can be considered that of personal influence. When the group benefit from influencing the individual pilot's behavior to conform to the “way they do things”, this direct pressure from peers is indicative of social influence. Such as, where groups are being rewarding for behaving in a certain way, even if that involves taking risks, new members will be expected to conform to the group norm.

Again, social influences are not always direct and the actions and behaviors of peers can influence individuals in ways that are less obvious, such as the informational social influence. Defined as the influence to accept information obtained from another as evidence of reality (50), in situations that are ambiguous, where the accuracy of the information is important and where others are considered experts, individuals are more likely to conform to informational social influence. In the case of VFR flight into IMC, the situation consists of all three factors, and thus, when a pilot knows a fellow pilot is flying in a specific location, they may believe the weather conditions are suitable for them. The influence is aptly described by the statement “The chief pilot [..] went. And I figured if he can do it–I can do it” (50).

**Organizational influence:** The perceptions of group norms and values, that determine the way individuals act and react to risk is considered to relate to organizational factors including safety culture (21). Two strong studies and one moderate study indicated associations related to organizational factors (36, 49, 50).

In the context of pilots being instructed to take-off and see how bad the weather is, and where this risky behavior leads to no negative consequences, the practice can become normalized by the group. The pilots become accustomed to the risk-taking because that is what's expected of them (49, 50). These cultural norms can become the organizational culture and the low organizational safety standards can lead to pilots being willing to take more risks and to reason away risky decisions (36). The incremental acceptance of progressively lower levels of safety by a group is the definition of the normalization of deviance (6).

#### Safety Motivation

Overall, the types of multilevel association identified in the empirical research show parallels to an individual's level of safety motivation, on a continuum from extrinsic to intrinsic motivation. For example, the associations related to social and organizational influences describe situations where the pilots might feel pressured to perform an activity and feels obligated to do it. The pilot's safety motivation is considered controlled and thus the associations demonstrate a source of extrinsic motivation.

Extrinsic motivation relies on the mechanism of obtaining reward or avoiding undesired outcomes (23). In general, accidents have a low likelihood of occurring, thus those engaging in this type of risk-taking behavior will likely observe little or no adverse consequence in undertaking the activity. Therefore, the extrinsic motivation for not engaging in the behavior, due to the avoidance of an undesired outcomes can become weakened. In fact, the incremental acceptance on this behavior by the pilot group, the normalization of deviance (6) could be argued as the internalization of the extrinsic motivation of obtaining reward, that of “getting the job done”.

### Limitations and Methodological Considerations

The articles reviewed, were retrieved in August 2019, thus it is possible that further research related to the subject of interest in this review has since been published. As such, due to the time frame between retrieval and submission, it is possible that important findings are absent from the analysis. Of note on 11 March 2020 the World Health Organization declared the COVID-19 outbreak a global pandemic. The impact the pandemic had on the aviation industry has been unprecedented. This review therefore provides a record of the risk-taking landscape prior to the impacts of the COVID-19 Pandemic, but the authors acknowledge that this landscape may be significantly different now.

As only published works were included in the review the results may have been subject to publication bias (65), however, the types of risk taking evaluated were consistent with that observed in Australian and New Zealand accident reports.

Another limitation of this review was that only three qualitative studies made the inclusion criteria, this potentially limited the degree to which the social and organizational factors could be explored. By their nature quantitative studies are proximal to their subject of interest and thus an “up and out” approach to contextualizing their findings is often limited in scope.

Considering the thematic nature of the synthesis, the types of study design of predominant use (cross-sectional and semi-structured interview) were deemed appropriate and considered not to impact the overall quality of this review.

This type of thematic analysis can however, have the potential to introduce biases from the researcher. Due to the thematic nature of this review, the findings are not intended to provide causal explanations, but to provide context that may further the understanding of multilevel influences that may be in effect. Little or no weight should be given to the number or relative frequency of the reported associations. This limitation is particularly important to keep in mind from a practical application of the research.

### Directions for Further Research

This review highlights the complex nature of multi-level influences related to VFR flight into deteriorating conditions / IMC. With more people turning to Part 135 type operations for their transport needs, due to the global pandemic, the key findings provide information for organization to consider as part of their risk management processes and safety management systems. Simply informing pilots of the probabilities, consequences and situations in which VFR into IMC accidents are likely is considered to be one means of reducing the risk associated with VFR flight in marginal weather (58). This review has however, highlighted that the influences on the pilot's decision-making and propensity to engaging in risk-taking during normal operations are multilevel. Focusing efforts solely on the pilot, in order to train the pilot, may well fall short of reducing the risk. Organization should thus direct focus on both social and organizational factors such as organizational norms or social pressures, which may have a significant influence on pilot risk-taking on a much broader scale.

Within the context of the pandemic aviation operations have had to comply with numerous additional requirements, for example, the requirement to refuse boarding if a passenger has COVID-19 type symptoms. Given the remote environments Part 135 pilots operate, a pilot could be pressured by a passenger presenting with COVID-19 type symptoms, to let them board, when the alternative is to leave them at the remote location. Furthermore, while the timeframe between retrieval of the articles and submission is acknowledge as a limitation, this review provides a basis from which future research can compare the risk landscape in Part 135 type operations. Future research should explore whether the types of risk-taking have changed post pandemic and if the multilevel influences identified in this review are also present within the COVID-19 pandemic context.

It was noted in this review that fatigue was only referenced by a single article (36), as a factor affecting pilots decision making. Common effects of fatigue are decreased visual acuity, singlemindedness, judgment errors, and indifference (66). These effects show similarities to associations identified, and in particular fatigue could play an interesting role in relation to the “reluctance to admit defeat / incur personal inconvenience” association. For example, a pilot may not want to admit that they are fatigued/too tired to carry on, and/or may not want to discontinue a flight, when there is limited choice for accommodation. This may lead to pilots continuing the flight when fatigued, potentially leading to further judgement errors. Outside of fatigue as a symptom, the risk of fatigue is considered to have increased due to the enduring impacts of the pandemic (67), and the management of fatigue is considered to be a shared responsibility of employees and employers (68). It is thus recommended that the role of fatigue in influencing risk-taking be further explored, both from an individual and organizational level perspective.

Considering the multilevel association identified by this review, parallels could be drawn to an individual's safety motivation, providing insight into the potential mechanism behind risk-taking in normal aviation operations. In addition, gaining further understanding of the multilevel influences and the potential relationships these have with an individual's self-determined and non-self-determined motivation, may provide insight into how organizations and government departments may better facilitate desired behaviors (51). Thus, future research should further investigate the individual, social and organizational factors that might influence the normalization of risk using an SDT lens. Shedding further light on the complexity of this phenomena and potentially offering means to mitigate the risk-taking motivation (both intrinsic and extrinsic), when decision-making involves a high degree of autonomy.

## Conclusion

Why pilots continue to fly VFR into deteriorating weather is considered an increasingly important but complex problem, with an extensive body of research suggesting that there are multiple factors at play. The unique contributions of this review include the potential for a multilevel influence approach to assist researchers and practitioners in understanding the nature of risk-taking behavior in normal aviation operations. specifically in terms of personal, social and organizational factors and the potential interrelationship with safety motivation. The studies in this review increase our overall understanding of multilevel influences on normalized risk-taking in the context of VFR flight into IMC. Across all findings in the 10 articles the following conclusions were made. All associations can be categorized under two overarching themes, being “continuation influence” and “acceptance of risk / normalization of deviance.” One or both themes can be consistently observed across the finding in all studies.

In general, this review points to the value of considering the social and organizational influences on normalized risk-taking and further exploring the association with safety motivation through an SDT lens. Although future studies must move beyond a narrow focus on pilot decision-making, and adopt a more holistic systems thinking methodology, there is ample evidence to suggest that the multi-level influences identified show linkages to the individuals intrinsic and/or extrinsic safety motivation.

Together, the findings of this review suggest that the reason why pilots engage in VFR flight into IMC could be related to safety motivation. As Goh and Wiegmann (60) state, “pilots may diagnose and perceive the risks accurately, but other motivational factors bias their decisions.” These motivational factors may be “continuation influences” or the desire to get the job done, or the fear of being ostracized by the group. These multilevel motivation influences may reflect group and cultural norms, where routine violation and risk-taking behavior are accepted and normalized as the “way things are done.” Furthermore, if the individual considered there to be no undesired outcome from the activity, then motivation to comply with the safety standards might well be lacking.

## Data Availability Statement

The original contributions presented in the study are included in the article/Supplementary Material, further inquiries can be directed to the corresponding author.

## Author Contributions

All authors listed have made a substantial, direct, and intellectual contribution to the work and approved it for publication.

## Conflict of Interest

The authors declare that the research was conducted in the absence of any commercial or financial relationships that could be construed as a potential conflict of interest.

## Publisher's Note

All claims expressed in this article are solely those of the authors and do not necessarily represent those of their affiliated organizations, or those of the publisher, the editors and the reviewers. Any product that may be evaluated in this article, or claim that may be made by its manufacturer, is not guaranteed or endorsed by the publisher.

## Footnotes

^1^Civil Aviation Rule (CAR) Part 135 (4) prescribes the operating requirements for air operations, air transport and commercial transport operations involving helicopters and small aeroplanes.

^2^Civil Aviation Rule (CAR) Part 135 defines small aeroplanes as having a passenger seating configuration of nine seats or less, excluding any required crew member seat, or a Maximum Certified Take Off Weight (MCTOW) of 5700 kg or less.

^3^Single engine instrument flight rules passenger operations are not eligible under CAR Part 135.

^4^To undertake CAR Part 135 type operations, pilots must hold at least a Commercial Pilot Licence (CPL).
