# Supplementary material for: A Systematic Review of Multilevel Influenced Risk-Taking in Helicopter and Small Airplane Normal Operations
Source: Front Public Health. 2022 May 12;10:823276. doi: 10.3389/fpubh.2022.823276 (PMC9133595; doi:10.3389/fpubh.2022.823276)
Supplement: Supplementary file 1 [file Table_1.DOCX]

**SLR Search Strategy and Included/Excluded Articles**

**SLR Research Questions**

**SLR the research questions posed are:**

1. What types of risk-taking behaviour do pilots operating within CAR Part 135 type operations engage in?
2. What are the influences/incentives that lead pilots to engage in risk-taking behaviour?

**Practical Screening Criteria**

**Inclusion Criteria:**

1. A focus on risk-taking - intentionally deviating from minimum standards (violation) or knowingly pushing ones’ own or the aircrafts safety limitations,
2. a focus on identifying variables that influence/predict/incentivise violation or risk-taking relating to aviation safety standards or regulations,
3. the participants were commercial pilots (held a commercial Pilot Licence (CPL)) or were operating under the requirements of CAR Part 135,
4. the article is available online,
5. the article was written in the English Language or an English version was available.

**Exclusion criteria:**

1. The subject matter related to an aeroplane with a seating capacity over nine seats, excluding crew member seats, and a maximum certified take-off weight of over 5700kg^[[1]](#footnote-1)^,
2. the subject matter related to single engine instrument flight rules passenger operations^2^,
3. the participants did not include commercial pilots, for example were solely non-pilots/student or trainee pilots/airline transport pilots/military pilots, and
4. the article does not relate to aviation or does not meet any of the inclusion criteria.

**Search Strategy – Academic Studies**

**Sources**

EBSCOhost E Journal, PsycArticles, PsycINFO

ScienceDirect

Taylor and Francis Online

SAGE

Springer

Library search
Unique References

**Databases**

Search updated 18 August 2019 All Databases and Library Query

***EBSCOhost E Journal -*** , PsycArticles, PsycINFO

Search Terms: Aviation AND (Pilot OR Aircrew OR Crew) AND ("Risk-Taking" OR “Risk Perception” OR “Risky Decisions” OR Viola* OR Unsafe Act* OR “Non-Compliance) NOT (Unmanned OR Military OR Army OR Navy OR Air-Force OR Defence) NOT (Health* OR Medic* OR Surgery)

Limits: Scholarly (Peer Reviewed) Journals, 1982 – 2019.

Search Results: 98

***ScienceDirect* –**

Safety Science, Accident Analysis & Prevention, European Journal of Operational Research, Reliability Engineering & System Safety, Transportation Research Part F: Traffic Psychology and Behaviour.

Search Terms: (Pilot OR Aircrew OR Crew) AND ("Risk-Taking" OR “Risk Perception” OR “Risky Decisions” OR Violation OR Unsafe Act OR “Non-Compliance)

Limits: Review Articles, Research Articles, Data Articles, Replication Studies, All Years.

Search Results: 1,856

***Taylor and Francis Online* –**

Search Terms: Aviation AND (Pilot OR Aircrew OR Crew) AND ("Risk-Taking" OR “Risk Perception” OR “Risky Decisions” OR Viola* OR Unsafe Act* OR “Non-Compliance) NOT (Health* OR Medic* OR Surgery)

Limits: Only Content I Have Full Access to. All Years.

Search Results: 344

***SAGE* –**

Search Terms: [[All aviation] OR [All helicopter] OR [All aircraft] OR [All aeroplane] OR [All "part 135"]] AND [[All pilot] OR [All aircrew] OR [All crew]] AND [[All "risk-taking"] OR [All "risk perception"] OR [All "risky decisions"] OR [All viola*] OR [All unsafe]] AND [[All act*] OR [All “non-compliance]] AND NOT [[All unmanned] OR [All military] OR [All army] OR [All navy] OR [All air-force] OR [All defence]] AND NOT [[All health*] OR [All medic*] OR [All surgery]]

Limits: Research Articles, 1924 – 2019.

Search Results: 190

***Springer* –**

Search Terms: Aviation AND (Pilot OR Aircrew OR Crew) AND ("Risk-Taking" OR “Risk Perception” OR “Risky Decisions” OR Viola* OR Unsafe Act* OR “Non-Compliance) NOT (Unmanned OR Military OR Army OR Navy OR Air-Force OR Defence) NOT (Health* OR Medic* OR Surgery

Limits: Article, All Years.

Search Results: 51

***USQ Library* –**

Search Terms: ("Risk-Taking" OR “Risk Perception” OR “Risky Decisions”) AND (Pilot OR Aircrew OR Crew) AND (Aviation OR Helicopter OR Aircraft OR Aeroplane OR “Part 135”) NOT (Unmanned OR Military OR Defence) NOT (Health* OR Medic* OR Surgery)

Limits: Exclude Newspaper Articles

Search Results: 302

&

Search Terms: (Viola* OR “Unsafe Act*” OR “Non-Compliance” OR “Deviance”) AND (Pilot OR Aircrew OR Crew) AND (Aviation OR Helicopter OR Aircraft OR Aeroplane OR “Part 135”) NOT (Unmanned OR Military OR Defence) NOT (Health* OR Medic* OR Surgery)

Limits: Articles, Conference Proceedings, Peer-Reviewed Journals, Available Online, All Years

Search Results: 1,901

Total titles screened = 4,742

Of total titles screened, No. of abstracts screened = 133

Breakdown of abstracts screened:

EBSCOhost E Journal, PsycArticles, PsycINFO = 32

ScienceDirect = 40

Taylor and Francis Online = 14

SAGE- Reviewed = 6

Springer- Reviewed = 0

USQ Library search = 37

Unique References = 4 (previously not seen)

Duplicates identified = 39

Total for practical screen = 94

No. excluded at practical screening = 57 (see excluded studies and reasons – Appendix B)

No. included for full text review = 37

Due to the specific context of the study being CAR Part 135 Pilot Risk-taking it was considered most appropriate to limit studies to only those engaging actual pilots and specifically CAR Part 135 eligible pilots in the final selection.

16 studies were therefore excluded at initial full text review due to study type (see excluded studies and reasons – Appendix C)

Studies reviewed and assessed against inclusion and exclusion criteria as a group = 21 Studies.

Studies excluded at group review = 11 (see excluded studies and reasons – Appendix D)

Studies that met inclusion and exclusion criteria and included in study = 10 (see included studies – Appendix A)

**Appendix A - List of included studies**

Bearman, C., Paletz, S. B. F., Orasanu, J. (2009). Situational Pressures on Aviation Decision Making: Goal Seduction and Situation Aversion. *Aviation, Space, and Environmental Medicine 80*(6)

Hunter, D. R., Martinussen, M., Wiggins, M., O’Hare, D. (2011). Situational and personal characteristics associated with adverse weather encounters by pilots. *Accident Analysis and Prevention 43*(1), 176–186

Michalski, D. J., Bearman, C. (2014). Factors affecting the decision making of pilots who fly in Outback Australia*. Safety Science 68*(1), 288–293

O'Hare, D., Smitheram, T. (1995). 'Pressing On' Into Deteriorating Conditions: An Application of Behavioral Decision Theory to Pilot Decision Making. *The International Journal of Aviation Psychology 5*(4), 351-370

Paletz, S. B. F., Bearman, C., Orasanu, J., Holbrook, J. (2009). Socializing the Human Factors Analysis and Classification System: Incorporating Social Psychological Phenomena Into a Human Factors Error Classification System. *Human Factors: The Journal of Human Factors and Ergonomics Society 51*(4), 435-445

Pauley, K., O'Hare, D. (2006). Measuring Risk Tolerance in General Aviation Pilots. *Proceedings of the Human Factors and Ergonomics Society Annual Meeting 50*(21), 2350-2353

Pauley, K. A., O'Hare, D., Mullen, N. W., Wiggins, M. (2008). Implicit Perceptions of Risk and Anxiety and Pilot Involvement in Hazardous Events. *Human Factors: The Journal of Human Factors and Ergonomic Society 50*(5), 723-733

Pauley, K., O'Hare, D., Wiggins, M. (2008). Risk tolerance and pilot involvement in hazardous events and flight into adverse weather. *Journal of Safety Research 39*(4), 403–411

Wiggins, M. W., Azar, D., Hawken, J., Loveday, T., Newman, D. (2014). Cue-utilisation typologies and pilots’ pre-flight and in-flight weather decision-making. *Safety Science 65*(1), 118–124

Wiggins, M. W., Hunter, D. R., O’Hare, D, Martinussen, M. (2012). Characteristics of pilots who report deliberate versus inadvertent visual flight into Instrument Meteorological Conditions. *Safety Science 50*(3), 472–477

**Appendix B - List of excluded studies and reasons for exclusion**

**Non-Aviation Context: n = 12**

Baldissonea, G., Combertia, L., Boscaa, S., Murèb, S. (2019). The analysis and management of unsafe acts and unsafe conditions. Data collection and analysis. *Safety Science 119(1)*, 240–251

Burt, C. D. B, Banks, M. D., Williams, S. D. (2014). Safety risks associated with helping others. *Safety Science 62*(1), 136–144

Hopkins, A. (2011) Risk-management and rule-compliance: Decision-making in hazardous industries. *Safety Science 49*(2), 110–120

Kansea, L., Parkesa, K., Hodkiewicza, M., Hua, X., & Griffin, M. (2018). Are you sure you want me to follow this? A study of procedure management, user perceptions and compliance behaviour. *Safety Science 101*(1), 19–32.

Lawton, R. (1998). Not working to rule: understanding procedural violations at work. *Safety Science Vol. 28*(2), 77–95

Nielsen, K. J., Hansen, C. D., Bloksgaard, L, Christensen, A., Jensen, S. Q., Kyed, M. (2015). The impact of masculinity on safety oversights, safety priority and safety violations in two male-dominated occupations. *Safety Science 76*(1), 82–89

Pandita, B., Alberta, A., Patila, Y., Al-Bayatib, A. J. (2019). Impact of safety climate on hazard recognition and safety risk perception. *Safety Science 113*(1), 44–53

Park, J., Jung, W. (2003). The operators’ non-compliance behavior to conduct emergency operating procedures—comparing with the work experience and the complexity of procedural steps. *Reliability Engineering and System Safety 82*(2), 115–131

Rimmo, P., Aberg, L. (1999). On the distinction between violations and errors: sensation seeking associations. *Traffic Psychology and Behaviour 2*(3), 151-166

Seo, D. (2005). An explicative model of unsafe work behaviour. *Safety Science 43*(3), 187–211.

Taylor, W. D., Snyder, L. A. (2017). The influence of risk perception on safety: A laboratory study. *Safety Science 95*(1), 116–124

Zhang, Z., Polet, P., Vanderhaegen, F., Millot, P. (2004). Artificial neural network for violation analysis. *Reliability Engineering and System Safety 84*(1), 3–18

**Non-Commercial Pilot/Non-Part 135 Context: n = 29**

**Focus on Airline Operations/Airline Pilots:**

Baksteen, B. (1995). Flying is not safe. *Safety Science 19*(2) 287-294

Carim, G. C., Saurin, T. A., Havinga, J, Rae, A., Dekker, S. W. A., Henriqson, É. (2016). Using a procedure doesn’t mean following it: A cognitive systems approach to how a cockpit manages emergencies. *Safety Science 89*(1), 147–157

Chen, J., Yu, V. F. (2018). Relationship between human error intervention strategies and unsafe acts: The role of strategy implementability. *Journal of Air Transport Management 69*(1), 112–122

Daramola, A. Y. (2014). An investigation of air accidents in Nigeria using the Human Factors Analysis and Classification System (HFACS) framework. *Journal of Air Transport Management 35*(1), 39-50

de Wit, P. A. J. M., Moraes Cruz, R. (2019). Learning from AF447: Human-machine interaction. *Safety Science 112*(1), 48–56

Denis Besnard, D., Greathead, D. (2003). A cognitive approach to safe violations. *Cognition, Technology & Work 5*(4), 272–282

English, D., & Branaghan, R. J. (2012). An empirically derived taxonomy of pilot violation behavior. *Safety Science, 50*(2), 199-209

Facci, E. L., Bell, M. A., Nayeem, R. (2005). The effect of social proof on weather-related decision making in aviation. *Proceedings of the human factors and ergonomics society annual meeting 49*(19), 1780-1784

Hale, A., Borys, D. (2013). Working to rule, or working safely? Part 1: A state of the art review. *Safety Science 55*(1), 207-221

Ji, M., You, X., Lan, J., Yang, S. (2011). The impact of risk tolerance, risk perception and hazardous attitude on safety operation among airline pilots in China. *Safety Science 49*(10), 1412–1420

Landry, S. J. & Jacko, J. (2012). Pilot Procedure-Following Behavior During Closely Spaced Parallel Approaches. *International Journal of Human-Computer Interaction 28*(2), 131-139

Low, J. M. W., Yang, K. K. (2018). An exploratory study on the effects of human, technical and operating factors on aviation safety. *Journal of Transportation Safety & Security*, 1-34

Matthews, R. A., Kauzlarich, D. (2000) The crash of valujet flight 592: a case study in state-corporate crime. *Sociological focus 3*(3), 281-298

Meng-Yuan, L. (2015). Safety Culture in commercial aviation: Differences in perspective between Chinese and Western pilots. *Safety Science 79*(1), 193–205

Plant, K. L., Stanton, N. A. (2012). Why did the pilots shut down the wrong engine? Explaining errors in context using Schema Theory and the Perceptual Cycle Model. *Safety Science 50*(1), 300–315

Shappell, S. A., Wiegmann, D. A. (1997). A Human Error Approach to Accident Investigation: The Taxonomy of Unsafe Operations. *The International Journal of Aviation Psychology 7*(4), 269-291

You, X., Ji, M., Han, H. (2013). The effects of risk perception and flight experience on airline pilots’ locus of control with regard to safety operation behaviors. *Accident Analysis and Prevention 57*(1), 131– 139

**Focus on Trainee/Cadets/Student/Non-Pilots:**

Alexander, M. (2013). Airplane Catastrophe as A Result of Operational Errors and Violations.
Aviation 17(2), 70-7

Brooker, P. (2005) Reducing mid-air collision risk in controlled airspace: Lessons from hazardous incidents. *Safety Science 43*(9) 715–738

Causse, M., Dehais, F., Péran, P., Sabatini, U., Pastor, J. (2013). The effects of emotion on pilot decision-making: A neuroergonomic approach to aviation safety. *Transportation Research Part C 33*(1), 272–281

Drinkwater, J. L., Molesworth, B. R.C. (2010). Pilot see, pilot do: Examining the predictors of pilots’ risk management behaviour. *Safety Science 48*(10), 1445–1451

Goh, J, Wiegmann, D. A. (2001). Visual Flight Rules Flight Into Instrument Meteorological Conditions: An Empirical Investigation of the Possible Causes. The international journal of aviation psychology 11(4), 359–379

Ji, M., Xu, Q., Xu, S., Du, Q., Li, D. (2018). Proactive personality and situational judgment among civil flying cadets: The roles of risk perception and cognitive flexibility. *Transportation Research Part F: Psychology and Behaviour 59*(1), 179–187

Ju, C., Ji, M., Lan, J., You, X. (2017). Narcissistic personality and risk perception among Chinese aviators: The mediating role of promotion focus. International Journal of Psychology 52(1), 1–8

Molesworth, B. R. C., Chang, B. (2009). Predicting Pilots’ Risk-Taking Behavior Through an Implicit Association Test. Human factors 51(6), 845-857

Molesworth, B.R.C, Tsanga, M. H., Kehoeb, E. J. (2011). Rehearsal and verbal reminders in facilitating compliance with safety rules. *Accident Analysis and Prevention 43*(3), 991–997

Molesworth, B. R. C., Wiggins, M. W. (2004). Improving pilots’ risk management behaviour through active involvement during training. *Proceedings of the human factors and ergonomics annual meeting 48*(22), 2553-2556

**Focus on Military Pilots:**

Harris, D., Li, W. (2019). Using Neural Networks to predict HFACS unsafe acts from the pre-conditions of unsafe acts. *Ergonomics 62*(2), 181-191

Thomson, M. E., O¨ nkal, D., lu, A. A., Goodwin, P. (2004). Aviation Risk Perception: A Comparison Between Experts and Novices. *Risk Analysis 24*(6), 1585-1595

**Non-Intentional Non-Compliance/Violation/Risk-Taking n = 16**

Chang, C. (2017). Risk factors associated with flying in adverse weather: From the passengers' point of view. *Journal of Air Transport Management 58*(1), 68-75

Changa, Y., Yangb, H., Hsiao, Y. (2016). Human risk factors associated with pilots in runway excursions. *Accident Analysis and Prevention 94*(1), 227–237

Chen, C., Chen, S. (2014). Measuring the effects of Safety Management System practices, morality leadership and self-efficacy on pilots’ safety behaviors: Safety motivation as a mediator. *Safety Science 62*(1), 376–385

Dorneich, M. C., Rogers, W., Whitlow, S. D., DeMers, R. (2016). Human Performance Risks and Benefits of Adaptive Systems on the Flight Deck. *The International Journal of Aviation Psychology 26*(1-2), 15-35

Gandera, P., Hartley, L., Powell, D., Cabond, P., Hitchcocke, E., Mills, A., Popking, S. (2011). Fatigue risk management: Organizational factors at the regulatory and industry/company level. *Accident Analysis and Prevention 43*(2), 573–590

Guo, Y., Sun, Y., Chen, S. (2018). Research on Human-Error Factors of Civil Aircraft Pilots Based On Grey Relational Analysis. *MATEC Web of Conferences 151*(1), 05005

Hartzler, B. M. (2014). Fatigue on the flight deck: The consequences of sleep loss and the benefits of napping. *Accident Analysis and Prevention 62*(10), 309– 318

Kelly, D., Efthymiou, M. (2019). An analysis of human factors in fifty controlled flight into terrain aviation accidents from 2007 to 2017. *Journal of Safety Research 69*(1), 155–165

Kontogiannis, T., Malakis, S. (2012). A systemic analysis of patterns of organizational breakdowns in accidents: A case from Helicopter Emergency Medical Service (HEMS) operations. *Reliability Engineering and System Safety 99*(16), 193–208

Li, G. (2003). Age, Flight Experience, and Risk of Crash Involvement in a Cohort of Professional Pilots. *American Journal of Epidemiology 157*(10), 874-880

Li, G., Bakera, S. P., Qianga, Y, Grabowskia, J. G., McCarthya, M. L. (2005). Driving-while-intoxicated history as a risk marker for general aviation pilots. *Accident Analysis and Prevention 37*(1), 179–184

Martin, D., Nixon, J. (2019) Helicopter pilots’ views of air traffic controller responsibilities: a mismatch. *Ergonomics 62*(2), 268-276

Moura, R., Beer, M., Patelli, E., Lewis, J. (2017). Learning from major accidents: Graphical representation and analysis of multi-attribute events to enhance risk communication. *Safety Science 99*(1), 58–70

Nascimentoa, F. A. C., Majumdara, A., Jarvis, S. (2012). Nighttime approaches to offshore installations in Brazil: Safety shortcomings experienced by helicopter pilots. *Accident Analysis and Prevention 47*(1), 64– 74

Pei, Y., Page, J., Pearce, G. (2017). Methodologies of stochastic simulation for helicopter accidents and nonparametric evaluation of the stochastic responses. International Journal of Crashworthiness 22(3), 332-346

Sunder, J., Sunder, S. V., Zhang, J. (2017). Pilot CEOs and corporate innovation. *Journal of Financial Economics 123*(1), 209–224

**Appendix C – List of studies excluded at initial full text review due to study type.**

**CAR Part 135 Context but Non-Participant Group Studies n = 16**

Baker, S. P., Lamb, M. W., Li, G., & Dodd, R. S. (1993). Human Factors in Crashes of Commuter Airplanes. *Aviation, Space, and Environmental Medicine 64*(1), 63-68.

Ballard, S. B., Beaty, L. P., & Baker, S. P. (2013). US commercial air tour crashes, 2000-2011: burden, fatal risk factors, and FIA Score validation. *Accident Analysis and Prevention 57*(1), 49-54.

Bazargan, M., Guzhva, V. S. (2011). Impact of gender, age and experience of pilots on general aviation accidents. *Accident Analysis and Prevention 43*(3), 962–970

Boyd, D. D., Stolzer, A. (2016). Accident-precipitating factors for crashes in turbine-powered general aviation aircraft. *Accident Analysis and Prevention 86*(1), 209–216

Burian, B. K., Orasanu, J., Hitt, J. (2000). Weather-Related Decision Errors: Differences across Flight Types. *Proceedings of the Human Factors and Ergonomics Society Annual Meeting 44*(1), 22-25

Cline, P. E. (2018). Human Error Analysis of Helicopter Emergency Medical Services (HEMS) Accidents Using the Human Factors Analysis and Classification System (HFACS). *Journal of Aviation/Aerospace Education & Research 28*(1).

Filho, A. P. G., Souza, C. A., Siqueira, E. L. B., Souza, M. A., Vasconcelos, T. P. (2019). An analysis of helicopter accident reports in Brazil from a human factors perspective. *Reliability Engineering and System Safety 183*(1), 39–46

Ison, D. (2014). Correlates of Continued Visual Flight Rules (VFR) into Instrument Meteorological Conditions (IMC) General Aviation Accidents. *Journal of Aviation/Aerospace Education & Research 24*(1)

Lenné, M. G., Ashby, K, Fitzharris, M. (2008). General Aviation Crashes in Australia Using the Human Factors Analysis and Classification System. *The International Journal of Aviation Psychology 18*(4), 340-352

Madhavan, P, Lacson, F. C. (2006). Psychological Factors Affecting Pilots’ Decisions to Navigate in Deteriorating Weather. *North American Journal of Psychology Vol. 8*(1)

Munene, I. (2016). An Application of the HFACS Method to Aviation Accidents in Africa. *Aviation Psychology and Applied Human Factors 6*(1), 33–38

Murray, S. R. (1999). FACE: Fear of Loss of Face and the Five Hazardous Attitudes Concept. *The International Journal of Aviation Psychology 9*(4), 403-411

Orasanu, J., Davison, J., Ciavarelli, A. Cohen, M., Fischer, U., Slovic, P. (2001). The Many Faces of Risk in Aviation Decision Making. *Proceedings of the Human Factors and Ergonomics Society Annual Meeting 45*(4), 307-310

Shappell, S., Detwiler, C., Holcomb, K., Hackworth, C., Boquet, A., & Wiegmann, D. A. (2007). Human Error and Commercial Aviation Accidents: An Analysis Using the Human Factors Analysis and Classification System. *Human Factors 49*(2), 227-242.

Von Thaden, T. L., Wiegmann, D. A., Shappell, S. A. (2006). Organizational Factors in Commercial Aviation Accidents. The International Journal of Aviation Psychology *16*(3), 239-261

Wilson, D. R., Sloan, T. A. (2003). VFR Flight Into IMC: Reducing the Hazard. *Journal of Aviation/Aerospace Education & Research 13*(1). doi: https://doi.org/10.15394/jaaer.2003.1567

**Appendix D - List of excluded studies at initial full text review and reasons for exclusion**

**Not specifically related to aviation** – (note; neither of the two aviation studies reviewed by the study met the inclusion and exclusion criteria of this study)

Alper, S. J., Karsh, B. (2009). A systematic review of safety violations in industry. Accident Analysis and Prevention 41(4), 739-754

**Not specifically focus on the identification of the variables that influence/incentivise risk taking, but a focus on the indicators/measures of the behaviour**

Hunter, D. R. (2005). Measurement of Hazardous Attitudes Among Pilots. *The International Journal of Aviation Psychology 15*(1), 23-43

Hunter, D. R. (2006). Risk Perception Among General Aviation Pilots. *The International Journal of Aviation Psychology 16*(2), 135-144

Pauley, K., O'Hare, D., Wiggins, M. (2009). Measuring Expertise in Weather-Related Aeronautical Risk Perception: The Validity of the Cochran-Weiss-Shanteau (CWS) Index. *The International Journal of Aviation Psychology 19*(3), 201-216

**No participants holding a Commercial Pilot Licence in group**

Causse, M., Baracat, B., Pastor, J., Dehais, F. (2011). Reward and Uncertainty Favor Risky Decision-Making in Pilots: Evidence from Cardiovascular and Oculometric Measurements. *Applied Psychophysiology and Biofeedback 36*(4), 231-242

Johnson, C. M., Wiegmann, D. A. (2011). Pilot Error During Visual Flight Into Instrument Weather: An Experiment Using Advanced Simulation and Analysis Methods. *Proceedings of the Human Factors and Ergonomics Society Annual Meeting 55*(1), 138-142

Wiegmann, D. A., Goh, J., O'Hare, D. (2002). The Role of Situation Assessment and Flight Experience in Pilots' Decisions to Continue Visual Flight Rules Flight into Adverse Weather. *Human Factors: The Journal of Human Factors and Ergonomics Society 44*(2), 189-197

**CAR Part 135 Context but Non-Participant Group Studies – Archival data**

Rebok, G. W., Qiang, Y., Baker, S. P., Mccarthy, M. L., Li, G. (2005). Age, Flight Experience, and Violation Risk in Mature Commuter and Air Taxi Pilots. *The International Journal of Aviation Psychology 15*(4), 363-374

**Not specifically related to the context of risk-taking being reviewed by this SLR**

Baker, S. P., Lamb, M. W., Grabowski, J. G., Rebok, G., Li, G (2001). Characteristics of General Aviation Crashes Involving Mature Male and Female Pilots. *Aviation, Space, and Environmental Medicine 72*(5), 447-452.

McMurtrie, K. J., Molesworth, B. R. C. (2017). The Variability in Risk Assessment Between Flight Crew. *International Journal of Aerospace Psychology 27*(3-4), 65-78

Simpson, P., Wiggins, M. (1999). Attitudes Toward Unsafe Acts in a Sample of Australian General Aviation Pilots. *The International Journal of Aviation Psychology 9*(4), 337-350

1. Aircraft and aircraft operations that meet these criteria are not eligible to operate in accordance with CAR Part 135. [↑](#footnote-ref-1)
